# Increased cardiovascular risk among cancer survivors presenting with chest pain

**DOI:** 10.1093/ehjopen/oeaf129

**Published:** 2025-10-07

**Authors:** Kobi Faierstein, Rotem Tal-Ben Ishay, Ranel Loutati, Lynn Idan, Ido Cohen, Tal Caller, Yaacov R Lawrence, Roy Raphael, Yovel Peretz, Dana Fourey, Haim Mayan, Noya Shilo, Amit Segev, Elad Maor

**Affiliations:** Leviev Cardiovascular Institute, Sheba Medical Center, Emek HaEla St. 2, Ramat-Gan 5262000, Israel; Gray Faculty of Medical and Health Science, Tel-Aviv University, Tel-Aviv 69978, Israel; Gray Faculty of Medical and Health Science, Tel-Aviv University, Tel-Aviv 69978, Israel; Department of Internal Medicine E, Sheba Medical Center, Ramat-Gan 5262000, Israel; Leviev Cardiovascular Institute, Sheba Medical Center, Emek HaEla St. 2, Ramat-Gan 5262000, Israel; Program Arrow for Medical Research, Sheba Medical Center, Tel Hashomer, Ramat-Gan 5262000, Israel; Leviev Cardiovascular Institute, Sheba Medical Center, Emek HaEla St. 2, Ramat-Gan 5262000, Israel; Gray Faculty of Medical and Health Science, Tel-Aviv University, Tel-Aviv 69978, Israel; Department of Internal Medicine E, Sheba Medical Center, Ramat-Gan 5262000, Israel; Leviev Cardiovascular Institute, Sheba Medical Center, Emek HaEla St. 2, Ramat-Gan 5262000, Israel; Gray Faculty of Medical and Health Science, Tel-Aviv University, Tel-Aviv 69978, Israel; Gray Faculty of Medical and Health Science, Tel-Aviv University, Tel-Aviv 69978, Israel; Department of Radiation Oncology, Sheba Medical Center, Ramat-Gan 5262000, Israel; Leviev Cardiovascular Institute, Sheba Medical Center, Emek HaEla St. 2, Ramat-Gan 5262000, Israel; Gray Faculty of Medical and Health Science, Tel-Aviv University, Tel-Aviv 69978, Israel; Department of Internal Medicine E, Sheba Medical Center, Ramat-Gan 5262000, Israel; Leviev Cardiovascular Institute, Sheba Medical Center, Emek HaEla St. 2, Ramat-Gan 5262000, Israel; Gray Faculty of Medical and Health Science, Tel-Aviv University, Tel-Aviv 69978, Israel; Gray Faculty of Medical and Health Science, Tel-Aviv University, Tel-Aviv 69978, Israel; Department of Internal Medicine E, Sheba Medical Center, Ramat-Gan 5262000, Israel; Gray Faculty of Medical and Health Science, Tel-Aviv University, Tel-Aviv 69978, Israel; Department of Internal Medicine E, Sheba Medical Center, Ramat-Gan 5262000, Israel; Leviev Cardiovascular Institute, Sheba Medical Center, Emek HaEla St. 2, Ramat-Gan 5262000, Israel; Gray Faculty of Medical and Health Science, Tel-Aviv University, Tel-Aviv 69978, Israel; Leviev Cardiovascular Institute, Sheba Medical Center, Emek HaEla St. 2, Ramat-Gan 5262000, Israel; Gray Faculty of Medical and Health Science, Tel-Aviv University, Tel-Aviv 69978, Israel

**Keywords:** Chest pain, Cardio-oncology, NSTEMI, Pulmonary embolism, Atrial fibrillation

## Abstract

**Aims:**

To examine the association between a personal history of cancer and the likelihood of a cardiovascular diagnosis among patients presenting with chest pain.

**Methods and results:**

We analyzed data from consecutive adult patients hospitalized with a primary diagnosis of chest pain between 2007 and 2022, excluding those with active cancer or ST-elevation myocardial infarction. Patients were categorized into two groups: cancer survivors and other patients. The primary outcome was a cardiovascular probable diagnosis, defined as a composite of non-ST-segment elevation myocardial infarction, pulmonary embolism, new-onset atrial fibrillation, or mortality within 30 days. The final cohort included 37 819 patients with a median age of 65 years (Q1–Q3: 55–75), of whom 24 644 (65%) were men. Among these, 1838 (5%) had a history of cancer. A multivariable logistic regression model demonstrated that cancer survivors were 70% more likely to reach the study primary endpoint compared with other patients (*P* < 0.001). A propensity score matching model consistently demonstrated that cancer survivors were 40% more likely to meet the study endpoint (95% CI 1.2–1.7, *P* < 0.001). Over a median follow-up of 4.3 years (Q1–Q3: 2.1–7.3), 7035 (19%) patients died. Kaplan-Meier survival analysis indicated a cumulative probability of death of 29% ± 22% for cancer survivors vs. 12% ± 9% for other patients (*P* < 0.001, Log rank).

**Conclusion:**

Among patients admitted to the hospital with chest pain, a personal history of cancer is independently associated with a significantly higher likelihood of receiving a final cardiovascular diagnosis.

## Graphical Abstract

**Graphical Abstract:**
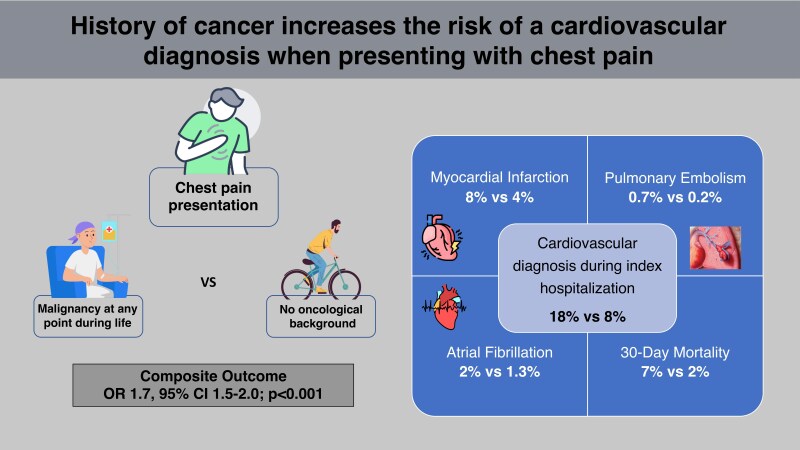

## Introduction

In recent decades, significant advancements in cancer treatment have led to improved patient outcomes and survival rates. However, as the oncology landscape continues to evolve, there is growing recognition that the journey toward cancer survivorship is not without challenges. One important challenge, often overlooked, is the emergence of cardiovascular complications in patients undergoing cancer therapy or living beyond their cancer diagnosis. As the population of cancer survivors continues to expand, it has become imperative to understand and address the complex relationship between cancer, cardiovascular risk factors, and adverse cardiovascular outcomes^1–3^ Chest pain is a common symptom that frequently prompts patients to seek medical care.^4^ However, its differential diagnosis spans a wide spectrum, ranging from life-threatening conditions such as pulmonary embolism and myocardial infarction to benign causes like musculoskeletal pain, thereby presenting a significant diagnostic challenge.^5^ The primary objective in managing patients with chest pain is to promptly exclude any life-threatening events. For this purpose, a comprehensive laboratory workup, including specific cardiac biomarkers, electrocardiogram (ECG) assessments, and point-of-care examinations, combined with the use of validated scoring systems, play a crucial role in directing the diagnosis. Various risk stratification score systems have been developed to aid in the assessment of chest pain patients. Nonetheless, their reliability in patients who have undergone cancer therapy remains unclear.^6^ As the population of cancer survivors grows, it becomes increasingly likely that they have undergone cancer therapeutics and radiation therapy, both of which carry the potential for toxic adverse effects on the cardiovascular system.^7^ While both traditional and novel cancer therapies have proven effective and offer hope from an oncological perspective, it is essential for physicians to consider the potential emergence of new morbidities resulting from these treatments.^8^ Cancer and cardiovascular diseases share common pathophysiologic risk factors, with inflammation being a major contributor.^9^ Furthermore, many commonly employed cancer treatments, including chemotherapy (e.g. doxorubicin), targeted agents (e.g. trastuzumab, bevacizumab), and radiation therapy, have significant cardiovascular side effects. Understanding the interplay between cancer therapy and cardiovascular health is vital for delivering comprehensive care to this unique and vulnerable patient population. Therefore, the purpose of the current analysis was to investigate how a personal history of cancer is associated with the likelihood of adverse cardiovascular outcomes among patients hospitalized with chest pain.

## Methods

### Study population

This retrospective cohort study included adults (≥18 years) presenting to the emergency department with chest pain between 2007 and 2022 at Sheba Medical Center, Israel’s largest hospital (∼115 000 annual admissions). Electronic medical records served as the primary data source. The institutional review board approved the study with waiver of individual consent due to strict anonymity protocols. Patients with ST-segment elevation myocardial infarction (STEMI) undergoing primary percutaneous coronary intervention (PCI) were excluded.

### Study definitions and endpoints

Patients were categorized into two mutually exclusive cohorts: (1) those with a history of cancer in remission and no oncologic therapy within six months of presentation, and (2) all other patients. Both solid and hematologic malignancies were included. Patients receiving active cancer treatment were excluded. Demographic and clinical baseline data were obtained from electronic medical records; cancer-related data and all-cause mortality were retrieved from national registries. Due to variability in assay protocols during the study period, troponin results were classified dichotomously (positive vs. normal). The primary outcome was a composite of cardiovascular events during the index hospitalization, including non-ST-segment elevation myocardial infarction, new-onset atrial fibrillation, pulmonary embolism, and 30-day mortality. The secondary outcome was all-cause mortality during follow-up.

### Statistical analysis

**Figure 1:**
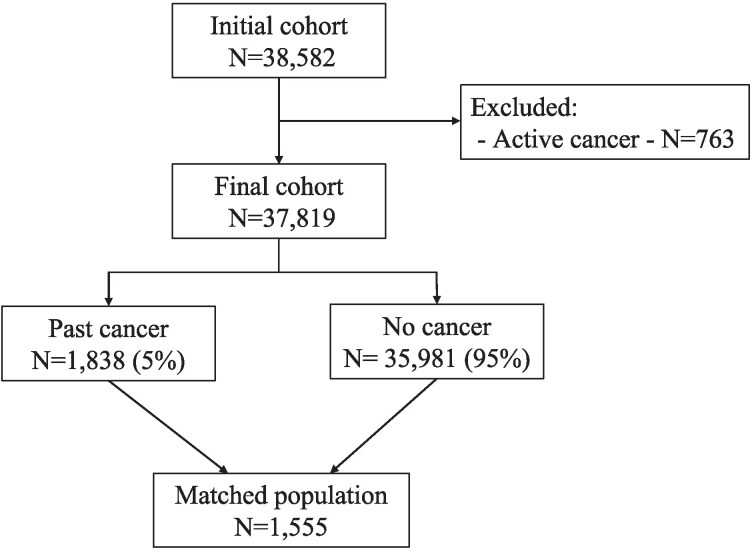
Study flowchart.

Binary logistic regression and propensity score matching models were used for data analysis. Continuous variables were reported as means with standard deviations (SD) if normally distributed, or as medians with interquartile ranges (Q1–Q3) if skewed. Categorical variables were presented as frequencies (percentages). Comparisons of continuous variables were performed using the Mann–Whitney U test, while categorical variables were compared using the Chi-square test or Fisher’s exact test. For the primary endpoint, additional covariates reflecting established cardiovascular risk factors; including age, heart failure, ischaemic heart disease (IHD), arterial hypertension, diabetes mellitus, chronic obstructive pulmonary disease (COPD), troponin levels, serum creatinine, and body weight, were incorporated into the logistic regression model. To minimize bias from imbalanced baseline characteristics, propensity score matching was performed using data from 1555 patients with complete clinical records. Matching accounted for age, heart failure, IHD, arterial hypertension, diabetes mellitus, COPD, troponin levels, serum creatinine, body weight, and serum sodium. Patients with incomplete clinical data were excluded from this analysis (*Figure 1*). For survival analyses, time to all-cause mortality was the dependent variable. The proportional hazards assumption was verified using Kaplan–Meier plots, which confirmed proportionality throughout follow-up. Survival probabilities with confidence intervals were estimated using the Kaplan–Meier method and compared across groups using the log-rank test. Univariable Cox proportional hazards regression was performed to calculate unadjusted hazard ratios (HRs) for all-cause mortality in patients with vs. without a cancer history. A multivariable Cox regression model was further adjusted for age, sex, IHD, heart failure, chronic kidney disease, COPD, atrial fibrillation, diabetes mellitus, arterial hypertension, serum troponin levels, and PCI during the index hospitalization. All analyses were performed using IBM SPSS Statistics for Windows, version 25.0.0 (IBM Corp., 2017) and R, version 4.3.0 (R Foundation for Statistical Computing). A two-sided *P*-value <0.05 was considered statistically significant.

## Results

The final cohort included 37 819 patients with a median age of 65 years (Q1–Q3: 55–75); 24 644 (65%) were men. A total of 1838 patients (5%) had a history of cancer. Compared with patients without cancer, those with an oncologic history were older (73 vs. 65 years) and had higher rates of comorbidities, including heart failure (14% vs. 6%), IHD (28% vs. 20%), atrial fibrillation (20% vs. 11%), chronic kidney disease (CKD) (14% vs. 6%), COPD (9% vs. 4%), hypertension (75% vs. 56%), diabetes mellitus (41% vs. 29%), and hyperlipidemia (46% vs. 32%; all *P* < 0.001). Nearly half (46%) of patients with a cancer history were admitted to the cardiology department, reflecting a higher index of suspicion at presentation. In contrast, only 11% were admitted to the dedicated chest pain unit compared with 25% of patients without cancer, consistent with their higher clinical risk profile. This was further supported by a greater prevalence of positive serum troponin levels among cancer patients (63% vs. 50%). In addition, patients with a cancer history were more likely to receive chronic cardiovascular medications (*Table 1*). The most prevalent cancer group consisted of hematologic and lymphoproliferative cancer types (*n* = 548), followed by gastrointestinal (*n* = 493), breast (*n* = 474), lung (*n* = 288) and, cancer of unknown origin (*n* = 35) (see Supplementary material online, *Table S2*).

**Table 1:** Baseline characteristics

		Oncologic background	
	All	No	Yes
	*n* = 37 819	*n* = 35 981 (95%)	*n* = 1838 (5%)
Age^a^	65 (Q1–Q3; 55–75)	65 (Q1–Q3; 54–74)	73 (Q1–Q3; 65–80)
Male	24 644 (65%)	23 741 (66%)	903 (49%)
Ischaemic heart disease	7564 (20%)	7054 (20%)	510 (28%)
Heart failure	2546 (7%)	2290 (6%)	256 (14%)
Atrial fibrillation	4185 (11%)	3819 (11%)	366 (20%)
COPD	1438 (4%)	1266 (4%)	172 (9%)
CKD	2345 (6%)	2090 (6%)	256 (14%)
Arterial hypertension	21 453 (57%)	20 074 (56%)	1379 (75%)
Diabetes mellitus	11 171 (30%)	10 418 (29%)	753 (41%)
Hyperlipidemia	12, 222 (32%)	11 371 (32%)	851 (46%)
Serum hemoglobin (g/dL)^a^	14 [12–15]	14 [12–15]	12 [11–14]
Serum troponin^b^ (ng/L)	60 [20–344]	61 [20–349]	52 [20–250]
PCI at index hospitalization	15 500 (41%)	14 700 (41%)	800 (44%)
Hospitalization ward:			
Internal medicine	11 137 (30%)	10 611 (30%)	726 (40%)
Cardiology	16 696 (44%)	15 848 (44%)	848 (46%)
ICU	437 (1%)	398 (1%)	39 (2%)
CPU	9037 (24%)	8838 (25%)	199 (11%)
Other	287 (1%)	285 (1%)	2 (0.1%)
Chronic cardiovascular pharmacologic therapy at index hospitalization:			
Antiplatelet medications	15 267 (40%)	14 087 (40%)	1180 (64%)
Anticoagulant agents	16 882 (45%)	15 526 (43%)	1356 (74%)
Beta-blockers	13 684 (36%)	12 758 (36%)	926 (50%)
Calcium-channel antagonists	7985 (21%)	7411 (21%)	574 (31%)
Angiotensin inhibitors^c^	15 020 (40%)	14 099 (39%)	921 (50%)
Statins	18 844 (50%)	17 720 (49%)	1124 (61%)

CKD, chronic kidney disease; COPD, chronic obstructive pulmonary disease; CPU, chest pain unit; eGFR, estimated glomerular filtration rate (was determined using the CKD Epidemiology Collaboration Creatinine Equation; ICU, intensive care unit; PCI, percutaneous coronary intervention

^a^Stands for mean values. Continuous variable presented as median [Q1–Q3], categorical variables presented as count (frequency).

^b^High-sensitivity troponin I.

^c^Angiotensin-converting enzyme inhibitors or angiotensin receptor blockers.

**Figure 2:**
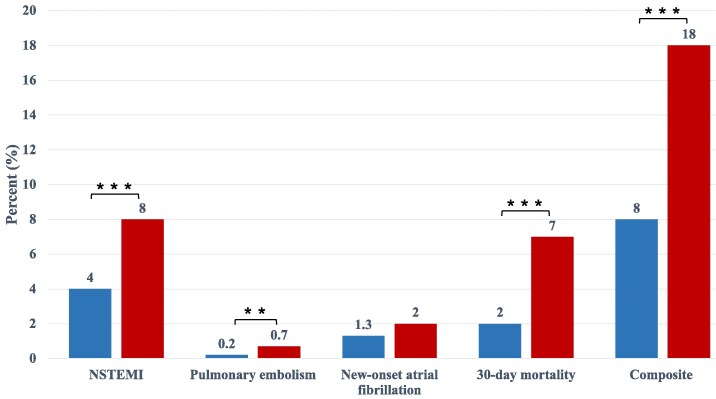
Bar chart demonstrates the composite outcome. Occurrence in percentage of the composite outcome and each component separately. Blue bars: non-oncologic group, red bars: oncologic group. The multivariable binary logistic regression model demonstrated an increased risk for a composite outcome in the oncologic group (OR = 1.7, 95% CI 1.5–2.0, *P* < 0.001). NSTEMI: non-ST-segment elevation myocardial infarction.

**Composite outcome:** A total of 3021 patients (8%) reached the composite outcome, including 322 (18%) with a history of cancer and 2699 (8%) without (*P* < 0.001). Significant differences were observed across all components of the composite outcome: non-ST-elevation myocardial infarction (NSTEMI) (8% vs. 4%), pulmonary embolism (0.7% vs. 0.2%), new-onset atrial fibrillation (2% vs. 1.3%), and 30-day mortality (7% vs. 2%) in patients with vs. without cancer history (*Table 2*). In multivariable binary logistic regression, cancer history was associated with a 70% increased risk of the composite outcome (OR 1.7, 95% CI 1.5–2.0; *P* < 0.001; *Table 3*, *Figure 2*). This effect was primarily driven by pulmonary embolism (OR 2.3, 95% CI 1.2–4.5; *P* < 0.001) and 30-day mortality (OR 2.1, 95% CI 1.7–2.6; *P* < 0.001). In univariable analysis, cancer history was associated with new-onset atrial fibrillation (OR 1.6, 95% CI 1.1–2.2; *P* < 0.001); however, this association was not significant in the multivariable model (OR 1.2, 95% CI 0.8–1.7; *P* = 0.41). Multivariable Cox regression similarly demonstrated an increased risk of the composite outcome in patients with cancer history (HR 1.7, 95% CI 1.5–2.0; *P* < 0.001; *Table 4*). Sex was not significantly associated with the composite outcome in either univariable or multivariable analyses, but was included in the models to adjust for potential confounding. A multivariable analysis according to cancer group demonstrated that among the known cancer origins, lung cancer had the highest odds ratio (OR = 2.8) to achieve the composite outcome. (see Supplementary material online, *Table S3*).

**Table 2:** Cardiovascular endpoints

		Oncologic background		
	All	No	Yes	*P* value
	*n* = 3021 (8%)	*n* = 2699 (8%)	*n* = 322 (18%)	
NSTEMI	1509 (4%)	1367 (4%)	142 (8%)	<0.001
Pulmonary embolism	101 (0.3%)	88 (0.2%)	13 (0.7%)	<0.001
New-onset AFib	490 (1.3%)	454 (1.3)	36 (2%)	0.01
30-day mortality	921 (2%)	790 (2%)	131 (7%)	<0.001

AFib, atrial fibrillation; NSTEMI, non-ST-segment elevation myocardial infarction.

Chi-square test for each endpoint by oncologic history.

**Table 3:** Association of cancer history with the composite outcome

	Univariable analysis			Multivariable analysis^a^		
	OR	95% CI	*P*-value	OR	95% CI	*P*-value
Composite outcome	2.6	2.2–2.9	<0.001	1.7	1.5–2.0	<0.001
NSTEMI	2.1	1.8–2.5	<0.001	1.4	1.2–1.7	<0.001
Pulmonary embolism	2.9	1.6–5.2	<0.001	2.3	1.2–4.5	0.011
New-onset AFib	1.6	1.1–2.2	0.011	—	—	—
30-day mortality	3.4	2.8–4.1	<0.001	2.1	1.7–2.6	<0.001

AFib, atrial fibrillation; CI, confidence interval; NSTEMI, non-ST-segment elevation myocardial infarction; OR, odds ratio.

^a^The model is further adjusted for age, sex, CKD, history of ischaemic heart disease, heart failure, chronic obstructive pulmonary disease, diabetes mellitus, arterial hypertension, serum troponin, percutaneous coronary intervention during index hospitalization, serum sodium levels.

Univariable and multivariable logistic regression models for all the baseline characteristics by oncologic history.

**Table 4:** Univariable and multivariable predictors for the composite outcome

	Univariable			Multivariable^a^		
	OR	95% CI	*P*-value	OR	95% CI	*P*-value
Oncologic background	2.6	2.2–2.9	<0.001	1.7	1.5–2.0	<0.001
Age	1.06	1.05–1.06	<0.001	1.04	1.03–1.04	<0.001
Male	1.0	0.9–1.1	0.49	0.9	0.8–1.0	0.131
CKD	3.6	3.2–4.0	<0.001	1.4	1.3–1.6	<0.001
Diabetes mellitus	2.2	2.0–2.4	<0.001	1.3	1.2–1.4	<0.001
History of AFib	1.5	1.4–1.7	<0.001	—	—	—
COPD	2.4	2.1–2.9	<0.001	1.4	1.2–1.6	<0.001
Heart failure	3.0	2.7–3.3	<0.001	1.3	1.1–1.5	0.001
History of IHD	2.5	2.3–2.8	<0.001	1.4	1.3–1.5	<0.001
Weight	0.993	0.990–0.995	<0.001	1.0	1.0–1.0	0.077

AFib, atrial fibrillation; CKD, chronic kidney disease; COPD, chronic obstructive pulmonary disease; IHD, ischaemic heart disease; PCI, percutaneous coronary intervention.

^a^The Multivariable model was further adjusted to serum sodium. Univariable and multivariable logistic regression models for all the baseline characteristics by oncologic history.

**Figure 3:**
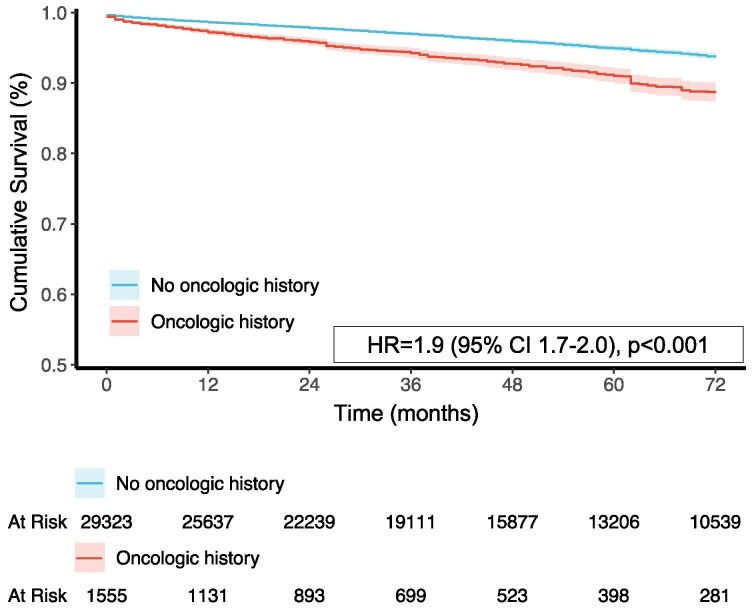
Adjusted cox survival analysis by oncologic background. Cox survival analysis adjusted for age, sex, diabetes mellitus, IHD, atrial fibrillation, congestive heart failure, COPD, CKD, troponin, PCI on admission and weight, depicting that patients with an oncologic background that were hospitalized for chest pain, were at an increased risk of death. Blue line: no oncologic background, red line: patients with an oncologic background.

**Figure 4:**
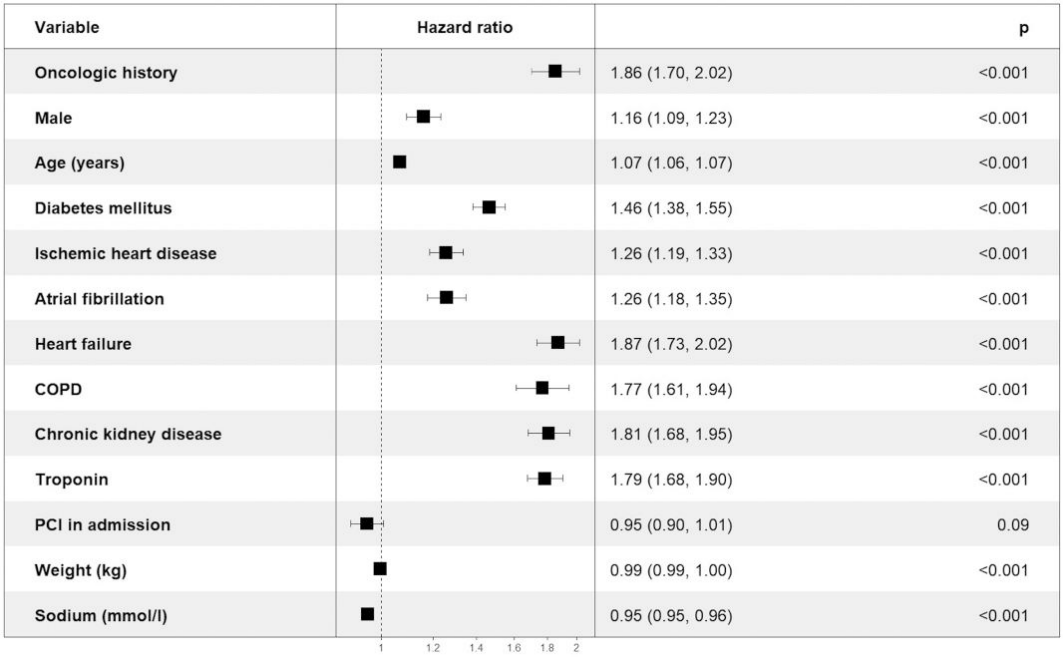
Multivariable COX regression model: time-to-all cause mortality by oncologic history and different independent variables. This Forrest plot describes the effect of each variable on the dependent variable, time to all-cause mortality. The hazard ratio, their 95% confidence interval and their significance (*P*) revealed that all variables are associated with all-cause mortality except for PCI in admission. COPD, chronic obstructive pulmonary disease; PCI, percutaneous coronary intervention.

**Mortality during follow-up:** During a median follow-up of 4.3 years (Q1–Q3: 2.1–7.3), 7035 patients (19%) died, including 712 (39%) with a history of cancer and 6323 (18%) without. Kaplan–Meier analysis demonstrated a higher cumulative probability of death at 4.3 years among patients with cancer history compared with those without (28.5% ± 21.6% vs. 12.0% ± 8.9%; log-rank *P* < 0.001). In multivariable Cox regression, a history of cancer was associated with a 90% higher risk of mortality during follow-up (HR 1.9, 95% CI 1.7–2.0; *P* < 0.001; *Figure 3*). Notably, even when accounting for established cardiovascular risk factors such as heart failure, CKD, and diabetes mellitus, cancer history remained a significant independent predictor of mortality (*Figure 4*). A multivariable analysis according to cancer group demonstrated that among the known cancer origins, lung cancer had the highest odds ratio (OR = 4.5) for all-cause mortality. (see Supplementary material online, *Table S4*).

**Propensity score matching:** To account for differences in group sizes, a 1:1 propensity score matching analysis was performed (see Supplementary material online, *Table S1* and *Figure S1*). After matching, no significant differences were observed in baseline characteristics, including age, sex, and cardiovascular risk factors, with standardized mean differences <10% for all variables. The matched analysis yielded consistent results, demonstrating an increased risk in patients with a cancer history for both mortality (HR 1.7, 95% CI 1.5–2.1; *P* < 0.001) and the composite outcome (OR 1.4, 95% CI 1.2–1.7; *P* < 0.001).

## Discussion

Our analysis has highlighted the significant association between a personal history of cancer and an elevated likelihood of experiencing cardiovascular diagnoses during hospitalization for chest pain; this includes non-ST-elevation myocardial infarction, pulmonary embolism, new-onset atrial fibrillation, and 30-day mortality. Previous studies consistently indicate that cancer survivors experience poorer outcomes and elevated mortality rates, underscoring the critical need for heightened awareness in clinical settings.^10–14^ This finding is particularly concerning given the global increase in cancer incidence, which is now the second leading cause of death worldwide.^15,16^

Ongoing advancements in diagnostic techniques and pharmacological therapies in oncology offer the potential for early diagnosis and more effective treatments, leading to prolonged survival and, in some cases, a cure. Nonetheless, it is well-documented that certain cancer therapies, including chemotherapy, targeted agents, and radiation therapy, are associated with cardiovascular toxicity and long-term adverse effects.^17–20^ Our analysis underscores the elevated risk of cardiovascular diagnoses among patients with a personal history of cancer admitted for chest pain, which can be partially attributed to the cardiotoxic effects of certain cancer therapies. Specifically, direct cardiotoxicity is observed with agents like anthracyclines, which can lead to structural damage in cardiac muscle cells.^21^ Furthermore, targeted therapies, such as tyrosine kinase inhibitors are associated with endothelial dysfunction and vasospasm, contributing to the increased incidence of cardiovascular events, such as acute myocardial infarction and hypertension. In addition, the chronic inflammation induced by these therapies can contribute to cardiovascular morbidity, as seen with immune checkpoint inhibitors that may trigger myocarditis.^22–25^ The metabolic effects of certain treatments, such as hormonal therapies, may also elevate cardiovascular risk. For instance, aromatase inhibitors can influence metabolic pathways, leading to hypertension, dyslipidemia, and insulin resistance, which are recognized risk factors for cardiovascular events.^26,27^ Lastly, for patients receiving radiation therapy, especially to the thorax, there is a well-documented risk of late cardiovascular complications due to radiation-induced damage to coronary arteries and the pericardium.^28,29^

Additionally, cancer and cardiovascular diseases share common pathophysiological mechanisms (e.g. chronic inflammation) and overlapping risk factors (e.g. diabetes mellitus, tobacco use, and obesity), which may contribute to increased cardiovascular morbidity among patients with cancer.^30–34^ Importantly, it is well known that cancer itself increases the risk of venous thromboembolism, particularly pulmonary embolism. Significantly higher proportion of patients in the cancer group were taking antiplatelet and anticoagulant medications compared with the non-cancer group. This observation is consistent with the clinical profile of cancer survivors, who often have a higher burden of cardiovascular comorbidities, such as IHD and atrial fibrillation. Notably, in our cohort, there is a higher prevalence of hypertension in the oncologic group (75% vs. 56%). These conditions could be either direct sequelae of cancer and its treatment or reflect the shared risk factors between cancer and cardiovascular disease, including chronic inflammation, metabolic syndrome, and smoking history. To address a potential confounding, we adjusted for relevant comorbidities and clinical variables in our multivariable logistic regression and propensity score matching analyses. The association between cancer history and adverse cardiovascular outcomes remained statistically significant after these adjustments, suggesting that the increased event rates are not solely explained by medication use or baseline comorbidity burden.

Up to 6.3% of emergency department visits are related to chest pain.^35^ Given the broad differential diagnosis, it is critical to promptly identify and address life-threatening conditions. Various risk scores, such as the HEART, GRACE, and TIMI, have been proposed for risk stratification,^36–38^ and contributing to the assessment of adverse cardiovascular events and death. Despite the high performance of the HEART score in predicting 30-day major adverse cardiovascular events (MACE), a prospective cohort study indicated that this score demonstrated low predictive ability in cancer survivors.^39–41^ Implementation of standardized screening tools that highlight patients’ cancer history can prompt healthcare providers to maintain a higher level of suspicion for potential cardiovascular diagnoses. Emergency department doctors may also benefit from establishing collaborative pathways with oncology services to facilitate prompt consultations for patients with a history of cancer. Moreover, integrating oncological considerations into existing risk stratification tools could further optimize care for this vulnerable group, ensuring they receive the specialized attention necessary during acute presentations.

Previous researches demonstrated increased cardiovascular mortality in cancer survivors.^42–44^

However, our study focused on cancer survivors that presented with chest pain, and consistently demonstrated an increased risk of both short- and long-term mortality. This may be partially explained by two factors: first, deaths occurring shortly after admission are more likely related to the acute presentation rather than pre-existing conditions, and can therefore plausibly be considered as cardiovascular mortality. Second, the long-term increased mortality among cancer survivors may be partially attributed to prior oncological treatments known to adversely impact cardiovascular function.

Advancements in oncology continue to improve, which leads to an early diagnosis and new therapeutic options. Malignancies that once considered life-threatening are now managed as chronic conditions, which results in increased life expectancy. This shift introduces a new population of cancer survivors who are at an elevated risk for cardiovascular morbidity. Preventive strategies for cardiovascular disease in cancer survivors are crucial for mitigating the elevated risk of cardiovascular morbidity in this population. Lifestyle interventions, regular physical activity, smoking cessation, and weight management, play a crucial role in reducing cardiovascular risk factors such as hypertension, dyslipidemia, and insulin resistance. Additionally, regular cardiovascular screenings, including blood pressure monitoring, lipid profiles, and echocardiograms, can help detect early signs of cardiac dysfunction and facilitate timely intervention. The involvement of cardiologists, particularly through dedicated cardio-oncology clinics, can ensure comprehensive care that integrates cancer treatment with vigilant cardiovascular risk management. Tailored monitoring and management plans can address the unique challenges faced by cancer survivors, ultimately enhancing their long-term health outcomes and quality of life. Integrating these preventive strategies into standard care for cancer survivors is essential for fostering better cardiovascular health in this at-risk group.

### Study strengths and limitations

This study has several important strengths. First, it is a large cohort of chest pain patients with and without cancer history who underwent comprehensive in-hospital evaluation. It spans over a very long period of time, and includes a variety of patients as Sheba Medical Center is a tertiary medical center in a single payer system that serves all the residents of Israel. By including four conditions in the composite endpoint, including 30-day mortality, we ensured that all patients with cardiovascular cause for their symptoms will be included in the analysis. Although the study was conducted in a small country, Israel is remarkably diverse, encompassing a broad spectrum of ethno-religious groups descendant from the Middle East, Europe, Africa (primarily North-East and Central East), Asia (mainly the Indian sub-continent), as well as North and South America. As Sheba is the largest tertiary medical center in the country, it attracts patients from all geographic areas in Israel, thus these groups are all appropriately represented in the sample with a substantial proportion of minorities and potentially socio-economically disadvantaged. Consequently, the study results are likely to have broader global applicability compared with many studies from Europe and even certain parts of North America.

Nevertheless, this study also has several limitations. It possesses all the inherent constraints of a retrospective, observational, single-center design. Additionally, our multivariable model did not incorporate certain important factors, such as ischaemic ST changes on the presenting ECG, which are critical in chest pain evaluation and may have led to residual confounding. Future prospective studies should incorporate standardized ECG collection to address this gap. Patients with chest pain who were discharged from the emergency department were also not included in the analysis. We did not differentiate between cancer types; instead, we included all patients with an oncologic history. Although the multivariable analysis demonstrated a significant statistical association between cancer types and study endpoints, the data appear to be limited due to a high prevalence of cancers of unknown origin, likely related to documentation issues. Furthermore, information was lacking for patients who received oncologic follow-up at a different medical center. We acknowledge that these factors may have introduced a degree of bias; however, the large-scale cohort remains a strength of this study. Finally, as we did not focus on the effects of different medications, and given the retrospective nature of the study, specific details about oncologic treatment were not available. Data on nicotine use were not available in our dataset, as this variable is inconsistently documented in electronic medical records. To partially mitigate this limitation, we incorporated COPD into the multivariable analysis, given its strong association with long-term smoking and its potential to serve as a surrogate marker for nicotine exposure. Nevertheless, we believe that future studies should be implemented with a focus on and comparison between different oncologic regimens. We must acknowledge the relatively high rate of long-term mortality of our cohort. We attribute this figure to the highly selected population that typically seek medical care, including patients with multiple comorbidities. We used body weight rather that BMI due to lack of this data and the lack of height for a proper calculation. Lastly, cause-specific mortality data were not available.

### Conclusions and clinical implications

The results could function to alert physicians to be more aware of the added risk of cardiovascular morbidity in patients with a history of cancer. A patient's personal history of cancer deserves particular attention when assessing chest pain. Our analysis demonstrates that patients with a history of cancer are more likely to have cardiovascular causes for their chest pain compared with the general population. Further research is needed to determine how incorporating cancer history could enhance risk assessment for patients presenting with chest pain.

## Supplementary Material

oeaf129_Supplementary_Data

## Data Availability

The data underlying this article will be shared on reasonable request to the corresponding author.
