# Supplementary material for: Increased cardiovascular risk among cancer survivors presenting with chest pain
Source: Eur Heart J Open. 2025 Oct 7;5(6):oeaf129. doi: 10.1093/ehjopen/oeaf129 (PMC12604472; doi:10.1093/ehjopen/oeaf129)
Supplement: oeaf129_Supplementary_Data [file oeaf129_supplementary_data.docx]

**Supplementary material**

**Table S1** – summary of balance data before and after propensity score matching

**A - Summary of Balance for All Data:**

|  | Means Treated | Means Control | Std. Mean Diff. | Var. Ratio | eCDF Mean | eCDF Max |
| --- | --- | --- | --- | --- | --- | --- |
| Distance | 0.08 | 0.05 | 0.59 | 1.48 | 0.21 | 0.32 |
| PCI in admission | 0.47 | 0.44 | 0.05 | . | 0.03 | 0.03 |
| Men | 0.51 | 0.67 | -0.32 | . | 0.17 | 0.16 |
| Age (years) | 71.85 | 64.25 | 0.65 | 0.71 | 0.08 | 0.28 |
| Diabetes mellitus | 0.42 | 0.30 | 0.25 | . | 0.12 | 0.12 |
| Ischemic heart disease | 0.28 | 0.21 | 0.16 | . | 0.07 | 0.07 |
| Atrial fibrillation | 0.20 | 0.11 | 0.23 | . | 0.09 | 0.09 |
| Heart failure | 0.14 | 0.07 | 0.22 | . | 0.08 | 0.08 |
| COPD | 0.09 | 0.04 | 0.19 | . | 0.05 | 0.05 |
| Chronic kidney disease | 0.15 | 0.06 | 0.25 | . | 0.09 | 0.09 |
| Troponin | 0.68 | 0.56 | 0.25 | . | 0.12 | 0.12 |
| Weight (kg) | 76.18 | 80.53 | -0.20 | 1.30 | 0.06 | 0.13 |
| Sodium (mmol/l) | 137.54 | 138.07 | -0.14 | 1.41 | 0.01 | 0.07 |

**B – Summary of Balance for Matched Data:**

|  | Means Treated | Means Control | Std. Mean Diff. | Var. Ratio | eCDF Mean | eCDF Max | Std. Pair Dist. |
| --- | --- | --- | --- | --- | --- | --- | --- |
| Distance | 0.08 | 0.08 | 0.000 | 1.0002 | 0 | 0.01 | 0.0003 |
| PCI in admission | 0.47 | 0.49 | -0.04 | . | 0.02 | 0.02 | 1.004 |
| Men | 0.51 | 0.52 | -0.02 | . | 0.01 | 0.01 | 0.81 |
| Age (years) | 71.85 | 72.55 | -0.06 | 0.93 | 0.01 | 0.04 | 0.71 |
| Diabetes mellitus | 0.42 | 0.44 | -0.04 | . | 0.02 | 0.02 | 0.90 |
| Ischemic heart disease | 0.28 | 0.29 | -0.03 | . | 0.01 | 0.01 | 0.90 |
| Atrial fibrillation | 0.20 | 0.20 | 0.005 | . | 0.002 | 0.002 | 0.67 |
| Heart failure | 0.14 | 0.14 | 0.02 | . | 0.01 | 0.01 | 0.58 |
| COPD | 0.09 | 0.08 | 0.05 | . | 0.02 | 0.02 | 0.46 |
| Chronic kidney disease | 0.15 | 0.13 | 0.05 | . | 0.02 | 0.02 | 0.52 |
| Troponin | 0.68 | 0.68 | 0.001 | . | 0.001 | 0.001 | 0.82 |
| Weight (kg) | 76.18 | 76.15 | 0.001 | 1.86 | 0.01 | 0.02 | 0.79 |
| Sodium (mmol/l) | 137.54 | 137.61 | -0.02 | 1.15 | 0.004 | 0.02 | 0.98 |

**C – sample sizes**

|  | Control | Treated |
| --- | --- | --- |
| All | 29323 | 1555 |
| Matched | 1555 | 1555 |
| Unmatched | 27768 | 0 |
| Discarded | 0 | 0 |

The data summary balance for all data and matched data demonstrated fair matching with a standard mean difference of less than 10% for all the baseline variables after matching.

**COPD:** chronic obstructive pulmonary disease, **Diff.:** difference, **eCDF:** empirical cumulative distribution function, **PCI:** percutaneous coronary intervention, **Std.:** standard deviation, **Var.:** variance,

**Figure S1 –** distribution of propensity scores and standardized mean difference of all data and of matched data

The left plot demonstrates a standardized mean difference, showing the balance of covariates

before and after matching. This is a visual assessment of the effectiveness of the matching

process in balancing the covariates between groups. The closer the "Matched" points are to

zero for each covariate, the better the balance achieved after matching. The vertical dashed

line at zero serves as a visual reference for balance. On the right - a density plot demonstrating the distribution of distance before and after a matching process categorized by an oncologic history. The matching process appears to have successfully reduced the difference in the distance distributions between individuals with and without an oncologic history. Therefore, suggests that the matching model effectively balanced the distance variable across the two groups.

**Table S2.** Frequencies of cancer types and treatments

|  | Frequency |
| --- | --- |
| Cancer origin: |  |
| Gastrointestinal n, (%) | 493 (1.3) |
| Stomach n, (%) | 45 (0.1) |
| Colon n, (%) | 383 (1) |
| Pancreas n, (%) | 29 (0.1) |
| Biliary n, (%) | 36 (0.1) |
| Breast n, (%) | 474 (1.3) |
| Lung n, (%) | 288 (0.8) |
| Hematologic and lymphoproliferative n (%) | 548 (1.5) |
| Lymphoma n (%) | 295 (0.8) |
| Leukemia n (%) | 166 (0.4) |
| Multiple myeloma n (%) | 87 (0.2) |
| Unknown origin n (%) | 35 (0.1) |
| Therapy applied: |  |
| Chemotherapy n (%) | 1170 (3.3) |
| Immune check point inhibitors n, (%) | 45 (0.1) |
| Radiation therapy n, (%) | 222 (0.6) |

Chemotherapy included different treatment protocols, all of whom consisted of at least one cardiotoxic agent (adriamycin, hydroxydaunorubicin, fluorouracil, platinum, and alkylating agents). Radiation therapy was included when involved radiation to the chest area.

**Figure S3:** univariable and multivariable analysis for composite outcome by cancer groups

|  | Univariable analysis | | | Multivariable analysis | | |
| --- | --- | --- | --- | --- | --- | --- |
|  | **OR** | **95% CI** | **P value** | **OR** | **95% CI** | **P value** |
| Gastrointestinal | 2.8 | 2.2-3.6 | <0.001 | 1.6 | 1.2-2.1 | <0.001 |
| Lymphoproliferative or hematologic | 2.5 | 2.0-3.1 | <0.001 | 1.6 | 1.3-2.1 | <0.001 |
| Breast | 1.9 | 1.4-2.5 | <0.001 | 1.4 | 1.0-1.9 | 0.05 |
| Lung | 4.0 | 3.0-5.4 | <0.001 | 2.8 | 1.9-3.9 | <0.001 |
| Unknown origin | 5.3 | 2.5-10.9 | <0.001 | 3.5 | 1.4-8.3 | <0.001 |

**Figure S4:** univariable and multivariable analysis for all-cause mortality by cancer groups

|  | Univariable analysis | | | Multivariable analysis | | |
| --- | --- | --- | --- | --- | --- | --- |
|  | **OR** | **95% CI** | **P value** | **OR** | **95% CI** | **P value** |
| Gastrointestinal | 3.3 | 2.7-3.9 | <0.001 | 1.6 | 1.3-2.0 | <0.001 |
| Lymphoproliferative or hematologic | 2.7 | 2.2-3.2 | <0.001 | 2.2 | 1.7-2.7 | <0.001 |
| Breast | 1.9 | 1.5-2.3 | <0.001 | 1.2 | 1.0-1.6 | 0.05 |
| Lung | 5.6 | 4.4-7.2 | <0.001 | 4.5 | 3.3-6.1 | <0.001 |
| Unknown origin | 3.9 | 2.0-7.7 | <0.001 | 2.1 | 0.9-5.1 | 0.09 |
